# Self-Assembly Vertical Graphene-Based MoO_3_ Nanosheets for High Performance Supercapacitors

**DOI:** 10.3390/nano12122057

**Published:** 2022-06-15

**Authors:** Ao Cheng, Yan Shen, Tianzeng Hong, Runze Zhan, Enzi Chen, Zengrui Chen, Guowang Chen, Muyuan Liang, Xin Sun, Donghang Wang, Linchen Xu, Yu Zhang, Shaozhi Deng

**Affiliations:** State Key Laboratory of Optoelectronic Materials and Technologies, Guangdong Province Key Laboratory of Display Material and Technology, School of Electronics and Information Technology, Sun Yat-sen University, Guangzhou 510275, China; chengao@mail2.sysu.edu.cn (A.C.); hongtz@mail2.sysu.edu.cn (T.H.); zhanrz3@mail.sysu.edu.cn (R.Z.); chenenz@mail2.sysu.edu.cn (E.C.); chenzr9@mail2.sysu.edu.cn (Z.C.); chengw57@mail2.sysu.edu.cn (G.C.); liangmy35@mail2.sysu.edu.cn (M.L.); sunx56@mail2.sysu.edu.cn (X.S.); wangdh26@mail2.sysu.edu.cn (D.W.); xulch3@mail2.sysu.edu.cn (L.X.); stszhyu@mail.sysu.edu.cn (Y.Z.); stsdsz@mail.sysu.edu.cn (S.D.)

**Keywords:** supercapacitors, vertical graphene, molybdenum trioxide, composite VG/MoO_3_ nanosheets, active sites

## Abstract

Supercapacitors have been extensively studied due to their advantages of fast-charging and discharging, high-power density, long-cycling life, low cost, etc. Exploring novel nanomaterial schemes for high-performance electrode materials is of great significance. Herein, a strategy to combine vertical graphene (VG) with MoO_3_ nanosheets to form a composite VG/MoO_3_ nanostructure is proposed. VGs as transition layers supply rich active sites for the growth of MoO_3_ nanosheets with increasing specific surface areas. The VG transition layer further improves the electric contact and adhesion of the MoO_3_ electrode, simultaneously stabilizing its volume and crystal structure during repeated redox reactions. Thus, the prepared VG/MoO_3_ nanosheets have been demonstrated to exhibit excellent electrochemical properties, such as high reversible capacitance, better cycling performance, and high-rate capability.

## 1. Introduction

Nowadays, environmental pollution has garnered increasing attention [1,2]. Green renewable energy storage systems have been fast developed instead of traditional non-renewable resources [3,4,5]. Among newly concerned energy storage devices, supercapacitors exhibit great advantages in fast-charging and discharging, high-power density, long-cycling lifetime, and low cost [6,7,8]. Electrode materials, as the most core part of supercapacitors, directly affect the overall performance of the devices. The supercapacitor electrode materials usually can be divided into two types, according to different energy-storage models [9]: One is carbon-based electrode (e.g., graphene [10], carbon nanotubes [11], porous carbon [12], etc.), that stores energy based on an electric double-layer capacitance theory. This form of storage relies on surface adsorption/desorption of ions and electrons; hence, the material structure of the electrode should be unchanged. The other is transition metal oxides-based electrode (e.g., MoO_3_ [13], Co_3_O_4_ [14], WO_3_ [15], MnO_2_ [16], etc.), that reserves energy through a pseudo capacitance mechanism. In this mechanism, ions and electrons are adsorbed on the surface of the active material or embedded in it and undergo redox reactions of surrounding materials to realize energy storage [17,18]. In general, the supercapacitor electrode materials require an excellent conductance, high reversible specific capacitance, long-term cycling stability, and large specific surface area when applied to actual energy storage devices.

Graphene stands out among carbon-based electrode materials due to its good electrical conductivity and large specific surface area formed by folds [19,20,21,22]. For such a two-dimensional nanostructure, the semi-metallic property is favorable to unimpeded electron transport and rapid electrochemical reaction kinetics, and the unique layered architecture provides adequate transmission paths for ions and electrons. Moreover, the large-area surface of graphene exhibits abundant active sites, which are beneficial for improving the electrochemical properties [23]. Graphene with a vertical growth orientation further exhibits a larger effective surface area that helps electrolyte infiltration [24]. However, the storage capacitance of pure graphene supercapacitors is generally considered inadequate [25]. In contrast, molybdenum trioxide (MoO_3_) is beneficial for a higher theoretical capacitance through redox reactions [26]. That makes it an important material for pseudo capacitance electrodes. However, the MoO_3_ electrode usually presents a poor electric conductivity and its volume and crystal structure are easy to change during the processes of charge and discharge, along with the variation of valence state [27,28,29]. All the disadvantages lead to a rapid decrease of storage capacitance and apparent irreversibility for the MoO_3_ electrode. The characteristics of graphene and MoO_3_ can be well complementary to synchronously obtain large capacitance, high conductance, and excellent cycling stability. For instance, Zhou et al., reported the preparation of MoO_3_-graphene aerogels (MoO_3_-GAs) via hydrothermal reaction. The MoO_3_-GAs were demonstrated to exhibit abundant exposed active sites, high specific capacitance (~527 F g^−1^ at 1 A g^−1^), and excellent cycling stability (~100% retention after 10,000 cycles) [30]. Yang et al., reported a graphene nanomesh-CNT/MoO_3−*x*_ (GC-MoO_3−*x*_) with three-dimensional sandwiched structure, which facilitated electrons and ions transport, and also exhibited high specific capacity up to ~427 F g^−1^ at 1 A g^−1^ [7]. Therefore, exploring novel and efficient graphene-based MoO_3_ composite nanomaterial electrodes is of great significance for developing high performance supercapacitors.

In this paper, a self-assembly vertical graphene-based MoO_3_ (VG/MoO_3_) nanosheet composite electrode was successfully prepared on nickel (Ni) foam by a two-step method successively growing VGs and then MoO_3_ nanosheets. The composite VG/MoO_3_ nanosheets exhibited improved electrochemical performances measured in ~1 mol L^−1^ Na_2_SO_4_ aqueous electrolyte in a three-electrode configuration. A high reversible cycling capacitance of 275 F g^−1^ was observed at the working current density of 1 A g^−1^, being approximately two-and-a-half times larger than the pristine VGs (~110.8 F g^−1^ at 1 A g^−1^). A high-rate ability of 80 F g^−1^ was also measured at a very large current density of 8 A g^−1^, which is five times higher than the pristine VGs and MoO_3_ nanosheets (~16 F g^−1^ at 8 A g^−1^). This is evidence that both of the electric-double-layer capacitance and pseudo-capacitance mechanisms contributed to the energy storage process in this case. The advantage of composites is further reflected in their interactions, particularly in the VGs, which act as a transition layer. Firstly, VGs perpendicularly grown on the Ni foam have a larger specific surface, not only providing more active sites for MoO_3_ nanosheet growth but also increasing adhesion between these active materials. Secondly, the VGs increase the property of electric contact between Ni foam and MoO_3_ nanosheets, which is beneficial for rapid and reversible redox reactions of MoO_3_. Thirdly, the stable VGs buffer the volume and crystal structure changes of MoO_3_ during the repeated charge and discharge operations, which helps to maintain the structural integrity of the electrode and to improve the cycling stability. The prepared VG/MoO_3_ nanosheets are demonstrated to be competitive candidates in electrode materials for high performance supercapacitors in the near future.

## 2. Materials and Methods

### 2.1. Materials

#### 2.1.1. Preparation of VGs

The synthesis of VGs was based on an inductively coupled plasma-enhanced chemical vapor deposition (ICPCVD) technique. A Ni foam was first ultrasonically washed with 1 mol L^−1^ H_2_SO_4_ for 10 min to remove the surface oxidation layer and was thereafter ultrasonically washed with ethanol and deionized H_2_O three times, respectively, to clean the surface. The Ni foam as a substrate was then put into the ICPCVD reaction chamber. After the vacuum of the system was pumped to 2.7 Pa by a mechanical pump, the substrate was heated to 800 °C and pretreated at H_2_ (~15 sccm) atmosphere with a radio frequency (RF) power of 900 W. Meanwhile a negative bias voltage (~100 V) was applied on the substrate to improve the plasma energy. After 15 min, the mixture of H_2_ (~10 sccm) and CH_4_ (~60 sccm) as a gas source was introduced into the reaction chamber for the growth of VGs. The growth reaction was performed at an RF power of 1100 W and a negative voltage of 100 V for 5 min. When the entire system was cooled down to room temperature, the growth of VGs on the Ni foam was completed.

#### 2.1.2. Preparation of VG/MoO_3_ Nanosheets

The VG/MoO_3_ nanosheets were thereafter prepared on the synthesized VGs using a modified thermal evaporation physical vapor deposition (PVD) method. The VGs were placed inside a bell jar chamber, keeping a certain distance with an evaporating molybdenum (Mo) boat source. The chamber was pumped to 10 Pa as a base vacuum by a mechanical pump. During the growth process, the Mo boat was first heated to 1000 °C at Ar (~150 sccm) atmosphere for 60 min, allowing large amounts of Mo oxide vapor to react and evaporate. Next, the temperature of the Mo source was dropped to 650 °C and then held at O_2_ (~10 sccm) and Ar (~150 sccm) atmosphere for 30 min (with a chamber vacuum of 65 Pa), to deposit and form the MoO_3_ onto the VG transition layer. Finally, the temperature of the system was decreased to room temperature to obtain the VG/MoO_3_ nanosheet product. For comparison, the pristine MoO_3_ nanosheets were synthesized in the same way directly using a blank Ni foam as the substrate.

### 2.2. Characterizations

The micro-morphologies of VG, MoO_3_, and VG/MoO_3_ nanosheets were observed by scanning electron microscopy (SEM, Supra 60, Zeiss, Jena, Germany) and transmission electron microscopy (TEM, Titan3 G^2^ 60–300, FEI Electron Optics B.V., Hillsboro, OR, USA). The analysis of material composition was conducted with energy dispersive spectrum (EDS) and corresponding mapping images, embedded in the SEM and TEM systems. The crystal structures of the samples were measured by powder X-ray diffraction (XRD, D-max 2200 VPC, Rigaku, Tokyo, Japan) and micro-Raman spectrometer (Raman, In Via Reflex, Renishaw plc, Gloucestershire, UK) with a 532 nm laser excitation. The valence states of elements in the samples were further analyzed by X-ray photoelectron spectroscopy (XPS, Escalab 250Xi, Thermo Fisher Scientific, Waltham, MA, USA).

### 2.3. Electrochemical Measurements

The electrochemical properties were measured with a three-electrodes system. The VG/MoO_3_, MoO_3_, and VG samples were respectively used as the working electrode, with a platinum (Pt) sheet (~10 mm × 10 mm) as the counter electrode and a saturated calomel as the reference electrode. The electrolyte was a solution of 42.6 g Na_2_SO_4_ in 300 mL deionized H_2_O. The electrochemical impedance spectroscopy (EIS), cyclic voltammetry (CV) curves, and galvanostatic charge-discharge (GCD) profiles of different electrode samples were measured in an electrochemical workstation (CHI660E, Shanghai Chenhua, Shanghai, China), with a testing voltage ranging from −1 V to −0.2 V.

## 3. Results and Discussion

**Figure 1:**
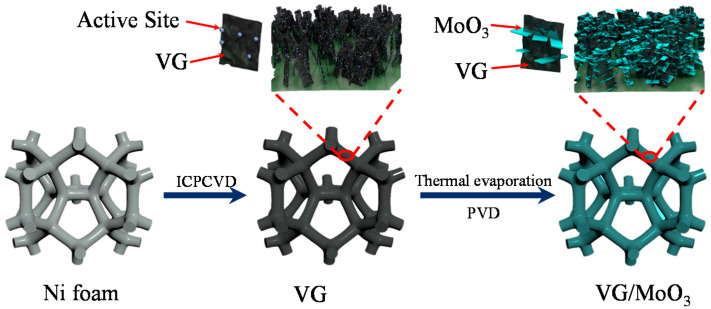
Schematic illustration for the synthesis process of composite VG/MoO_3_ nanosheets.

Our strategy for the synthesis of nanocomposite electrodes is to use the pre-grown VGs as a skeleton to further grow MoO_3_ nanosheets. Figure 1 shows the schematic illustration of the proposed synthesis strategy for self-assembly VG/MoO_3_ nanosheets. The VG/MoO_3_ nanosheets were prepared by a simple two-step method. Firstly, a ICPCVD approach was used to synthesize VG nanosheets on a Ni foam substrate, which acted as the current collector for supercapacitors. The surface of VGs should carry a large number of active sites. Afterward, MoO_3_ nanosheets were grown by a thermal evaporation PVD process, particularly originating from the active sites on the VGs’ surface. In the composite nanosheets, the existence of VGs is believed to improve the adhesion, electric contact, and structural stability of electrode materials for an excellent electrochemical performance. To demonstrate this, the pristine VGs and MoO_3_ nanosheets prepared directly onto the Ni foams were applied as the references for comparison with the VG/MoO_3_ nanosheets.

**Figure 2:**
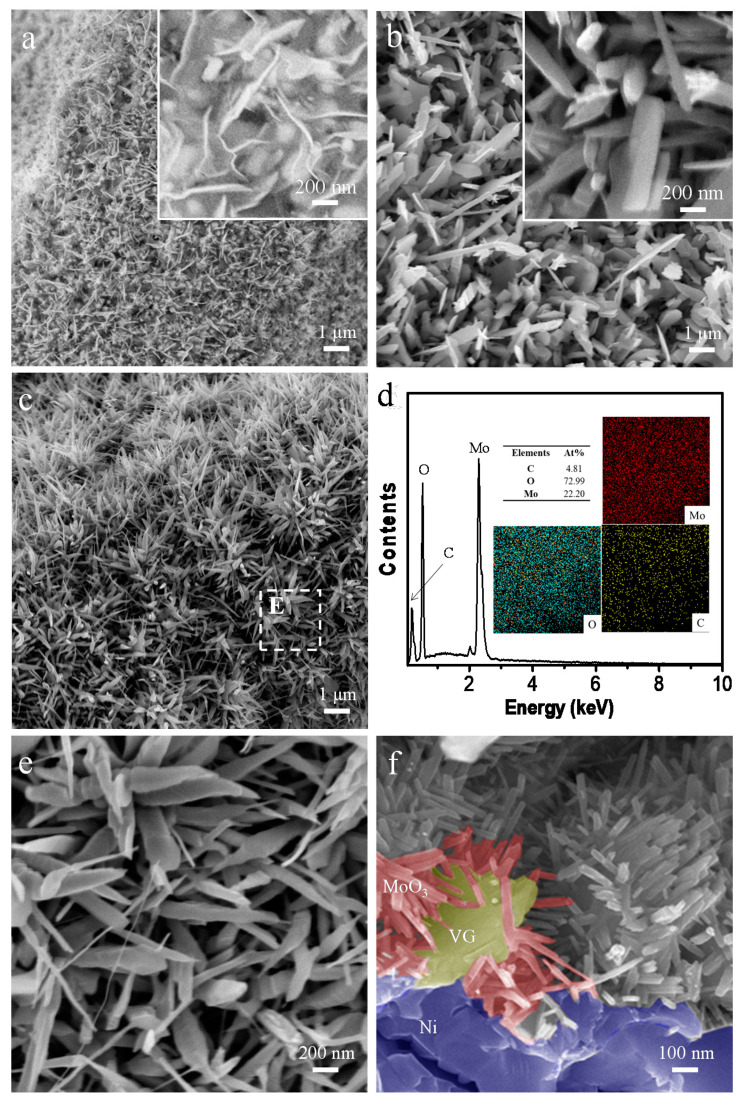
Micro-morphologies and material compositions of the prepared VG, MoO_3_, and VG/MoO_3_ samples. (**a**,**b**) SEM images of the pristine VGs and MoO_3_ nanosheets. Insets: high-magnification images of local areas. (**c**) Low-magnification SEM image of the prepared VG/MoO_3_ nanosheets. (**d**) EDS spectrum of the VG/MoO_3_ nanosheets. Insets: the corresponding EDS mappings of the three elements Mo, O, and C for (**c**) and their atomic percentages. (**e**) High-magnification SEM image of the marked area E in (**c**). (**f**) Typical cross-sectional SEM image of the VG/MoO_3_ nanosheets, in which some of the individuals are colored to highlight structural relationships.

The micro-morphologies and elemental compositions of the prepared VG, MoO_3_, and VG/MoO_3_ samples were characterized by SEM and EDS techniques. Figure 2a,b show typical SEM images of the pristine VGs and MoO_3_ nanosheets. The VGs were observed to distribute uniformly on the Ni foam substrate, typically being with a width of 500–600 nm and a thickness of approximately 20 nm. A more detailed SEM image and corresponding EDS mapping analysis (Figure S1, Supporting Information) demonstrated an existence of some Ni nanoparticles at the interface between the VGs and the substrate. Nickel has been proved to be an efficient catalyst to prepare carbon materials. Herein, the Ni particles generated in the ICPCVD process are believed to be the active sites for the growth of VGs. The pristine MoO_3_ nanosheets also exhibit a uniform distribution over the Ni foam, being with smooth surface and good crystallization. Further EDS analysis (Figure S2, Supporting Information) indicated that the atomic percentages of Mo and O were 23.31% and 76.69%, respectively, nearly to the ratio of 1:3. Figure 2c shows typical low-magnification SEM images of the prepared VG/MoO_3_ nanosheets. It was observed that in the composite sample, the distribution of nanosheets relied significantly on the cluster-shape of bottom VGs, implying that the VG structure with large-area surface acted as a skeleton for the subsequent MoO_3_ nanostructures growth. EDS analysis of the VG/MoO_3_ nanosheets (Figure 2d) shows homogeneous distributions of the three elements Mo, O, and C. No signals of Ni were detected, indicating the composite nanomaterial has completely covered the electrode surface. From the atomic percentages of the three elements, the content of C was measured as very slight, owing to its relative position closer to the bottom of the electrode than MoO_3_. From a high-magnification SEM image (Figure 2e) of the marked area E in Figure 2c, we can observe that the MoO_3_ nanosheets formed in the composite typically present smaller structural dimensions compared with the pristine ones (see Figure 2b). Herein, the MoO_3_ nanosheets are believed to originate from the sufficient active sites on the VG surface rather than the Ni foam, resulting in a smaller individual size and denser distribution. A typical cross-sectional SEM image of the VG/MoO_3_ nanosheets (Figure 2f) more clearly reveals the structural relationship between VGs and MoO_3_ nanosheets in the prepared composites, which is consistent with the proposed nanostructure model (see Figure 1).

**Figure 3:**
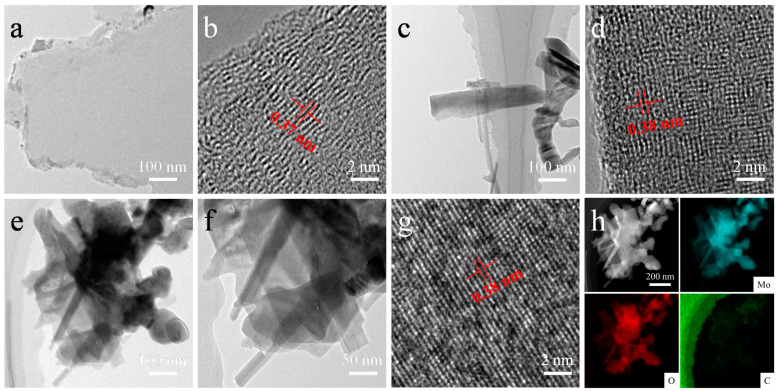
TEM characterizations of the prepared VG, MoO_3_, and VG/MoO_3_ samples. (**a**,**b**) Low-magnification image and HRTEM image of a typical VG structure. (**c**,**d**) Low-magnification image and HRTEM image of a typical MoO_3_ nanosheet. (**e**–**g**) Low-magnification images and HRTEM image of a typical VG/MoO_3_ nanosheet. (**h**) HADDF image and elemental mapping images of different elements Mo, O, and C existing in the VG/MoO_3_ structure.

More details in materials were employed in TEM, XRD, Raman, and XPS characterizations. As shown in Figure 3, TEM images of the typical VG, MoO_3_, and VG/MoO_3_ individuals were investigated. The low-magnification TEM image of the pristine VG presents a thin sheet-like structure (Figure 3a). In its edge position from the HRTEM image (Figure 3b), it is observed that the discontinuous lattice fringes are with a mean inter-planar lattice spacing of 0.37 nm, which corresponds to a few adjacent layers of graphene with defects [31,32]. EDS mappings (Figure S3, Supporting Information) show that a small amount of oxygen (O) was present in the VG surface. The existing O is most likely responsible for the defects in graphene, which can match to the active sites on the VG surface. Figure 3c,e show low-magnification TEM images of the MoO_3_ and VG/MoO_3_ nanosheets under the same scale. The pristine MoO_3_ has a homogeneous distribution of elements Mo and O in the entire structure (Figure S4, Supporting Information). In addition, both of the MoO_3_ nanostructures exhibit a sheet like structure, but the nanosheet grown on VGs is much smaller than that on Ni foam, which is in agreement with the SEM results. We believe that the active sites induced by the defects on the VG surface can act as the origins of the growth of MoO_3_ nanosheets. Consequently, the prepared VG/MoO_3_ nanosheet presents a structure of the bottom VG skeleton in combination with the upper MoO_3_ nanosheets (Figure 3f). The VG here helps to increase the number density and specific surface area of MoO_3_ nanosheets, thus increasing the reaction sites for ions transport and storage. Notably, the presence of graphene did not affect the intrinsic crystal structures of MoO_3_. HRTEM images (Figure 3d,g) show that both of the pristine MoO_3_ and composite VG/MoO_3_ nanosheets exhibit clear lattice fringes with an inter-planar spacing of 0.38 nm corresponding to (110) planes of MoO_3_. To investigate the distributions of Mo, O, and C in the VG/MoO_3_ composite, the HADDF image and corresponding EDS mapping images were also obtained (Figure 3h). It is observed that both of the elements Mo and O are distributed uniformly as that in the MoO_3_ nanosheet sample. However, the element C (see green spots in Figure 3h) is mainly distributed at the bottom side of the MoO_3_ nanosheet (the signals of sample were shielded to some extent by the carbon supporting film on the TEM copper grid), which corresponds to the VG transition layer of composite structure.

**Figure 4:**
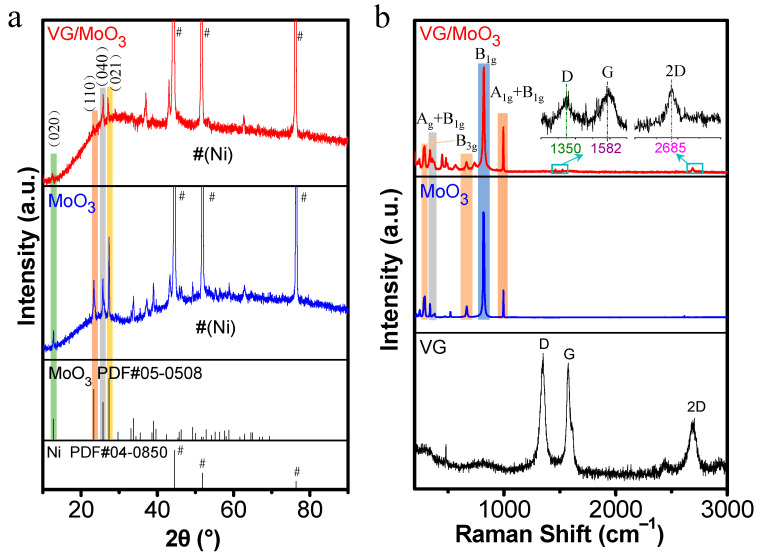
XRD and Raman characterizations of the prepared VG, MoO_3_, and VG/MoO_3_ samples. (**a**) XRD patterns of the pristine MoO_3_ and VG/MoO_3_ nanosheets (The symbol # represents the peaks of nickel foam). (**b**) Raman spectra of the three samples.

Figure 4a shows typical XRD patterns of the pristine MoO_3_ and composite VG/MoO_3_ nanosheets samples. The MoO_3_ nanosheets film exhibits a series of main diffraction peaks at 2θ = 12.7°, 23.3°, 25.7°, and 27.3°, which correspond to (020), (110), (040), and (021) crystal planes of α-MoO_3_ phase (JCPDS PDF#05-0508). Except for the diffraction peaks of α-MoO_3_ phase and those originated from the Ni foam (JCPDS PDF#04-0850, marked by symbol #), no characteristic peaks of other phases were observed, indicating that the synthesized material is pure MoO_3_. In addition, the composite VG/MoO_3_ nanosheets exhibit weaker intensities in the main characteristic peaks of α-MoO_3_ but with a quite apparent wave packet around approximately 2θ = 25°. This is attributed to the presence of VGs, which may contain some amorphous body of carbon.

Figure 4b shows the Raman spectra of the prepared VG, MoO_3_, and VG/MoO_3_ samples. The pristine VGs present two strong main peaks at 1350 and 1580 cm^−1^, corresponding to the D and G modes of carbon material, respectively. Specifically, the D peak can be identified as a structural defect-induced mode, while the G intensity is related to the graphitization degree. The sample also exhibits a secondary 2D characteristic peak at approximately 2700 cm^−1^, which indicates a certain degree of crystalline carbon material in the prepared VGs. In addition, the pristine MoO_3_ and the composite VG/MoO_3_ nanosheets exbibit similar vibration mode peaks of MoO_3_. The peak at 338 cm^−1^ is ascribed to the A_g_ + B_1g_ mode of O-Mo-O. The peaks of 292 and 667 cm^−1^ are derived from the B_3g_ mode, corresponding to the O-Mo-O and Mo-O-Mo band vibration, respectively. The peaks at 819 and 993 cm^−1^ are attributed to the B_1g_ mode of Mo-O-Mo band and A_1g_ + B_1g_ mode of O=Mo band, respectively [33,34]. In addition to the Raman peaks of α-MoO_3_ phase vibration modes, the composite VG/MoO_3_ nanosheets also exhibit very weak D, G, and 2D peaks that correspond to carbon material (see the inset localized enlarged spectra), being only with slight shifts compared with the pristine VG sample. This result proves that the VG transition layer still retained its original phase and crystal structure after undergoing the high-temperature growth process of the MoO_3_ nanosheets.

**Figure 5:**
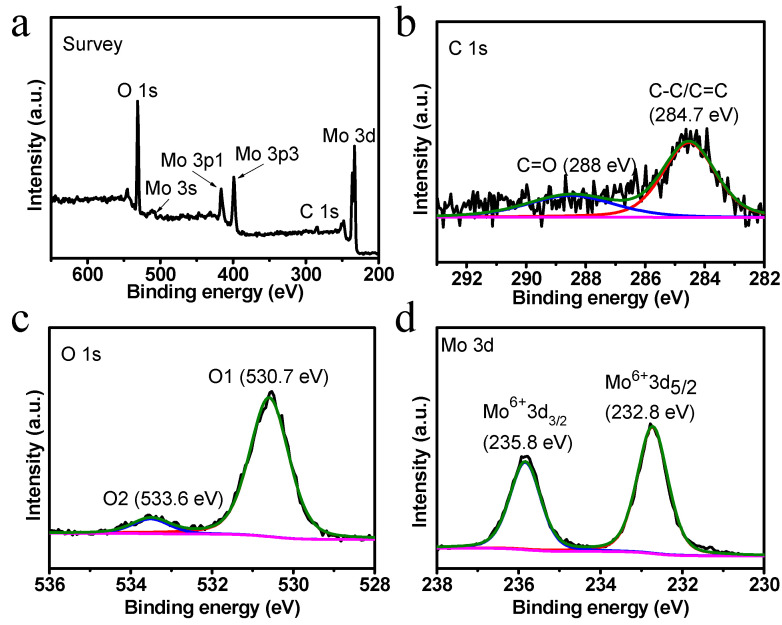
XPS characterization of the prepared VG/MoO_3_ nanosheets sample. (**a**) Wide-scanning survey XPS spectrum. (**b**–**d**) High-resolution XPS spectra of C 1s, O 1s, and Mo 3d in VG/MoO_3_, respectively.

The chemical composition and valence state of the prepared VG, MoO_3_, and VG/MoO_3_ samples were thereafter investigated by XPS. A wide-scanning survey spectrum of the VG/MoO_3_ nanosheets is shown in Figure 5a, revealing the existence of Mo, O, and C elements. To be more detailed, the high-resolution XPS spectrum of C 1s (Figure 5b) reveals characteristic peaks at 284.7 and 288 eV, which correspond to the C-C/C=C and C=O binding energy, respectively. The detecting intensities of C 1s in VG/MoO_3_ are significantly weaker than that in the pristine VGs (Figure S5, Supporting Information). This is because XPS is a surface analysis technique with a detecting depth of several nanometers; hence, the C 1s signal at the transition layer of the composite VG/MoO_3_ structure is difficult to detect. Next, the high-resolution spectra of O 1s and Mo 3d (Figure 5c,d) show that the concerned peaks of VG/MoO_3_ are almost identical to pure MoO_3_ (Figure S5, Supporting Information). On one hand, the O 1s spectrum displays two peaks at approximately 530.7 and 533.6 eV, which are assigned to the lattice oxygen (O1) and defects oxygen (O2), respectively. The former O1 is typically present in the MoO_3_ crystals, while the latter O2 is present in free oxygen on the surface of MoO_3_ or defects oxygen of VGs [35]. On the other hand, the Mo 3d spectrum shows two peaks at 232.8 and 235.8 eV, being consistent with the Mo^6+^ 3d^5/2^ and Mo^6+^ 3d^3/2^ states, respectively [36]. This accurately confirms the existence of the Mo^6+^ state of the composite nanostructures.

**Figure 6:**
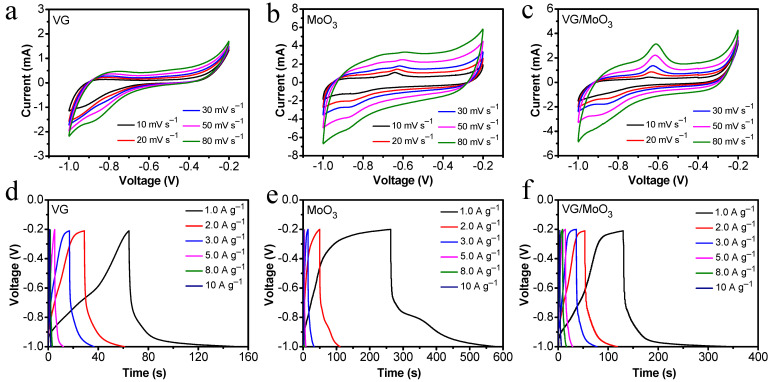
Electrochemical properties of the prepared VG, MoO_3_, and VG/MoO_3_ samples, with a testing voltage ranging from −1 V to −0.2 V. (**a**–**c**) CV curves at different scanning rates from 10 mV s^−1^ to 80 mV s^−1^. (**d**–**f**) GCD curves at different current densities from 1 A g^−1^ to 10 A g^−1^.

Now, we focus on the energy storage properties of different samples in a three-electrode measuring system to demonstrate the structural advantages of the composite VG/MoO_3_ nanosheets. Figure 6a–c show the cyclic voltammetry (CV) curves of the prepared VG, MoO_3_, and VG/MoO_3_ samples under different scanning rates ranging from 10 mV s^−1^ to 80 mV s^−1^. With an increase in scanning rate, the CV curves of all three samples showed varying degrees of increase in the testing current and corresponding CV area of the entire three-electrode system. It was observed that the recorded CV curves of the pristine VGs are with no redox peaks, indicating a charge/discharge behavior of ideal electric-double-layer model (Figure 6a). In contrast, with a scanning rate of 10 mV s^−1^, the CV curve of the pristine MoO_3_ nanosheets clearly shows an anodic peak at −0.64 V, implying an existing oxidation-reduction reaction process based on a pseudo-capacitance mechanism (Figure 6b). In addition, the intensity of the observed anodic peak decreased with an increase in sweep speed, being with a slight shift to the right. It can be explained that the poor conductivity of pristine MoO_3_ limits the rate of electrochemical redox reaction at high scanning rates. This will result in a poor rate performance of MoO_3_-based supercapacitors. However, after composited with VGs, the measured CV curves of the VG/MoO_3_ nanosheets exhibit more and more obvious anodic peaks as the sweep speed increased from 10 mV s^−1^ to 80 mV s^−1^ (Figure 6c). This demonstrates a rapid redox reaction capability, that facilitates ions and electrons transfer for a high-rate performance. The existing transition-layered VGs are believed to help increase the electric conductivity of the composite electrode material, which further provides abundant paths for ions and electrons to transfer and transport.

**Figure 7:**
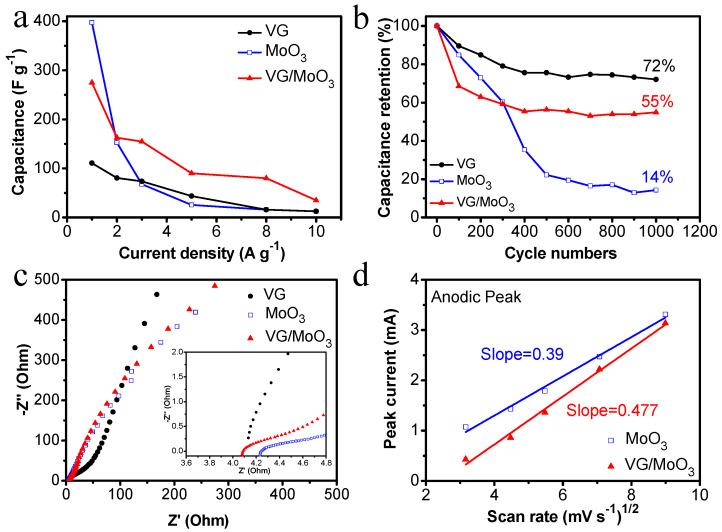
Comparison of electrochemical properties of the prepared VG, MoO_3_, and VG/MoO_3_ samples. (**a**) Rate performance. (**b**) Cycling performance at a current density of 2.0 A g^−1^. (**c**) Nyquist plots. Inset: high-frequency region of Nyquist plots. (**d**) Relationship between the anodic peak current (*i*_p_) and square root of scanning rate (*v*^1/2^) of the pristine MoO_3_ and composite VG/MoO_3_ nanosheets.

Figure 6d–f show the galvanostatic charge/discharge (GCD) properties of the three samples at different testing current densities. The discharging capacitance of the pristine VGs was measured as 110.8, 80.5, 73.8, 43.7, 16, and 12.5 F g^−1^ with the working current densities of 1, 2, 3, 5, 8, and 10 A g^−1^. Under these conditions, the discharging capacitance of the pristine MoO_3_ nanosheets was 397.5, 152.7, 67.8, 25.6, 16, and 12.5 F g^−1^. The specific rate performance of the samples is shown in Figure 7a to make the comparison easier. Notably, the VGs possess small capacitance at low current densities, but exhibit no dramatic degradation with an increase in current density, demonstrating a high-rate performance (black curve in Figure 7a). In contrast, the MoO_3_ nanosheets have large initial capacitances but a faster decay (blue curve in Figure 7a). The composite VG/MoO_3_ nanosheets combine the advantages of the aforementioned two materials. Hence, this electrode material presented a discharging capacitance of 275, 162.5, 154.8, 90, 80, and 35 F g^−1^ at different current densities of 1, 2, 3, 5, 8, and 10 A g^−1^, exhibiting both an excellent energy storage capability and a high-rate performance (see Figure 6f and red curve in Figure 7a). Such an outstanding rate capability of the VG/MoO_3_ sample is attributed to its unique VG transition layer structure, which provides plenty of high-conductivity paths for the transfer and diffusion of ions and electrons, and further improves the speed of electrochemical reactions.

The cycling capacitance retention rate is another important property for supercapacitors. Figure 7b shows the cycling performance of the prepared samples, repeatedly charging/discharging for 1000 cycles at a current density of 2.0 A g^−1^. The values of capacitance retention were recorded as 72%, 14%, and 55%, respectively, for the VG, MoO_3_, and VG/MoO_3_ samples. According to the electric-double-layer theory, the material structure or phase of the VG electrode did not change during the charging and discharging process, resulting in a preferable cycling stability. However, the capacitance retention of the pristine MoO_3_ nanosheets decreased rapidly with an increase in the process of charging/discharging. This is because the volume and crystal structure of pseudo-capacitive materials are easy to change in repeated redox reactions, which impairs the stability of electrode materials. Based on this, in the proposed VG/MoO_3_ nanosheets, the VG transition layer not only enhances the adhesion of MoO_3_ nanosheets but also relieves the volume and structure changes of electrodes. Therefore, the VG/MoO_3_ sample exhibited a better capacitance retention and cycling performance than the MoO_3_ electrode.

Electrochemical impedance spectroscopy (EIS) properties were also tested to study the electrochemical kinetics of different samples (Figure 7c). Typical Nyquist plots can be divided into two parts: the semicircle in high-frequency regions and the inclined line in low-frequency regions, commonly revealing the characteristics of interfacial resistance (*R*_i_), charge-transfer resistance (*R*_ct_), and Warburg impedance (*Z*_w_). Specifically, *R*_i_ is obtained from the intercept of semicircular start point and horizontal axis and it denotes the total resistance of electrodes, electrolytes, current collectors, etc. *R*_ct_ represents the diameter of the semicircle, referring to the charge accumulation/release resistance on the surface of electrode. In this study, the VG/MoO_3_ sample exhibited a relatively lower *R*_i_ of 4.09 Ω compared with the MoO_3_ sample (~4.25 Ω). This attributes to a reduced contact resistance with the assistance of VG transition layers between Ni foam and MoO_3_ nanosheets. However, the pristine VGs exhibited a similar value of *R*_i_ but a significant increase in *R*_ct_ compared with the other two materials, indicating difficult charge accumulation and release for pure graphene. In addition, the slope of inclined lines in Figure 7c is represented as *Z*_w_, which is related to the diffusion resistance inside the electrode. Herein, the pristine VGs show a steeper slope in low-frequency region, implying a fast diffusion of ions and electrons in the material. Unlike VGs, the other two samples reflect similar low-frequency characteristics and slopes. Therefore, we further studied the diffusion kinetic process of the MoO_3_ and VG/MoO_3_ samples by analyzing the CV curves (Figure 6b,c) under different scanning rates. According to the Randles–Sevcik theory [37,38,39], the anodic peak current (*i*_p_) can be described as follows:

*i*_p_ = (2.69 × 10^5^) *n*^3/2^ × *S* × *D*^1/2^ × *C* × *v*^1/2^ (1)

where *n* is the number of transferring electrons in the process of charge/discharge reactions, *S* is the effective contact area, *D* is the diffusion coefficient, *C* is the concentration of electrolyte, and *v* is the scanning rate. Since the values of *n*, *S*, and *C* are relatively constant, the anodic peak current (*i*_p_) and the square root of the scanning rate (*v*^1/2^) should have a linear relationship, in which the slope should be the diffusion coefficient (*D*). As shown in Figure 7d, the diffusion coefficient of the VG/MoO_3_ nanosheets (~0.477) was calculated to be greater than that of the MoO_3_ nanosheets (~0.39). The result indicates a rapid diffusion rate and superior rate capability of the composite nanomaterial, owing to the involvement of graphene carbon materials.

According to the above results, we believe that the energy storage performance of the proposed nano-scaled VG/MoO_3_ electrode should contain both behaviors of capacitive and diffusion-charging storage processes, which were controlled by the electric-double-layer capacitance mechanism (from VGs) and the pseudo-capacitance mechanism (from MoO_3_), respectively. With the speed of the scanning rate increasing, the capacitive behavior gradually becomes dominant. This is because the electrons and ions may have no time to diffuse to the deep layer of the electrode material for a reversible electrochemical reaction, which only occurs on the surface or near the surface of the material. Energy storage in such a case mainly depends on the adsorption of ions and electrons. Compared with MoO_3_, the VG/MoO_3_ nanosheets have smaller structural dimensions to increase the effective contact area, as well as lower charge transfer resistance and larger diffusion coefficient to accelerate ions and electrons transfer. Therefore, the VG/MoO_3_ electrode exhibits higher capacitive and diffusion-charging contributing behaviors at a high scanning rate.

## 4. Conclusions

In conclusion, to develop high performance electrode materials suitable for supercapacitors, we prepared vertical graphene-based MoO_3_ (VG/MoO_3_) nanosheets on Ni foam by a simple two-step self-assembly growth method. In the proposed composite nanomaterial, the VGs prepared in advance were used as the skeleton for the subsequent growth of MoO_3_ nanosheets, thus significantly improving the energy storage properties. Compared with the pristine VGs and the MoO_3_ nanosheets, the VG/MoO_3_ nanosheets exhibited high reversible capacitance (~275 F g^−1^ at 1 A g^−1^), better cycling performance (with a capacitance retention of 55% at 2 A g^−1^ after 1000 cycles), and high-rate capability (~80 F g^−1^ at 8 A g^−1^). The superior electrochemical performance is attributed to the VG transition layer inside the nanostructure, which was demonstrated to supply abundant surface active sites for the MoO_3_ nanosheets growth, increasing the specific surface area of MoO_3_. The existence of VGs also improves the conductivity of electrode material and stabilizes the volume and crystal structure change of MoO_3_ during repeated charging and discharging operations. Our study provides a simple but effective route to develop high-performance supercapacitors, particularly to improve long cycle stability performance for devices based on transition metal oxides.

## Data Availability

The data that support the findings of this study are available from the corresponding author upon reasonable request.

## Supplementary Materials

The following supporting information can be downloaded at: https://www.mdpi.com/article/10.3390/nano12122057/s1, Figure S1: Micro-morphologies and material compositions of the pristine VGs; Figure S2: Micro-morphologies and material compositions of the pristine MoO_3_ nanosheets; Figure S3: TEM HADDF image and elemental mapping images of the pristine VG; Figure S4: TEM HADDF image and elemental mapping images of the pristine MoO_3_ nanosheet; Figure S5: XPS characterizations of the pristine VGs and MoO_3_ nanosheets.
