# Supplementary material for: Self-Assembly Vertical Graphene-Based MoO3 Nanosheets for High Performance Supercapacitors
Source: Nanomaterials (Basel). 2022 Jun 15;12(12):2057. doi: 10.3390/nano12122057 (PMC9228046; doi:10.3390/nano12122057)
Supplement: Supplementary file 1 [file nanomaterials-12-02057-s001.zip › nanomaterials-1757441-supplementary.pdf]

nanomaterials

Supplementary Materials

Self-Assembly Vertical Graphene-Based MoO_3 Nanosheets for High Performance Supercapacitors

Ao Cheng, Yan Shen *, Tianzeng Hong, Runze Zhan, Enzi Chen, Zengrui Chen, Guowang Chen, Muyuan Liang, Xin Sun, Donghang Wang, Linchen Xu, Yu Zhang and Shaozhi Deng

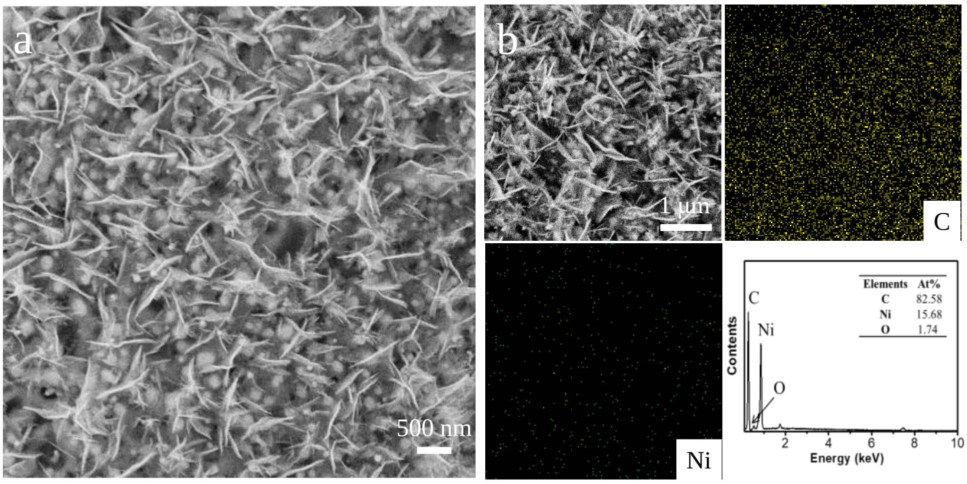

Figure S1. Micro-morphologies and material compositions of the pristine VGs. (**a**) Low-magnification SEM image. (**b**) EDS spectrum of the VGs and elemental mapping images of C and Ni in the sample.

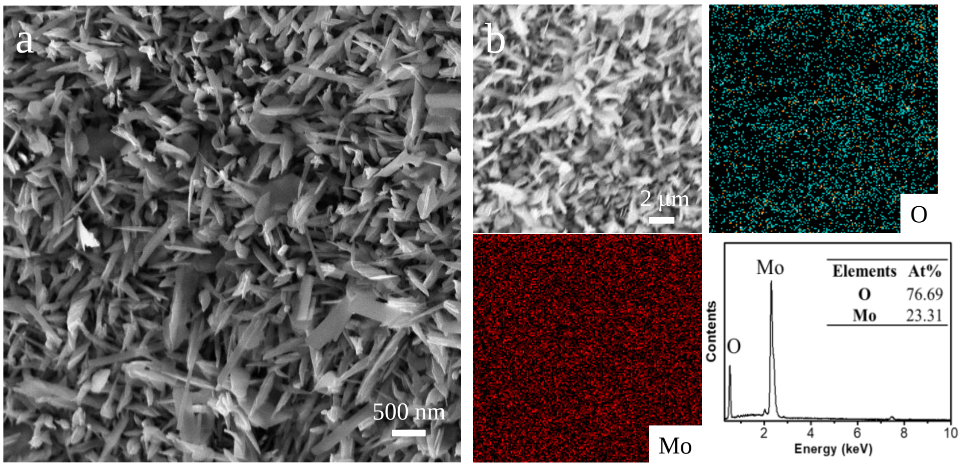

Figure S2. Micro-morphologies and material compositions of the pristine MoO_3 nanosheets. (**a**) Low-magnification SEM image. (**b**) EDS spectrum of the MoO_3 nanosheets and elemental mapping images of Mo and O in the sample.

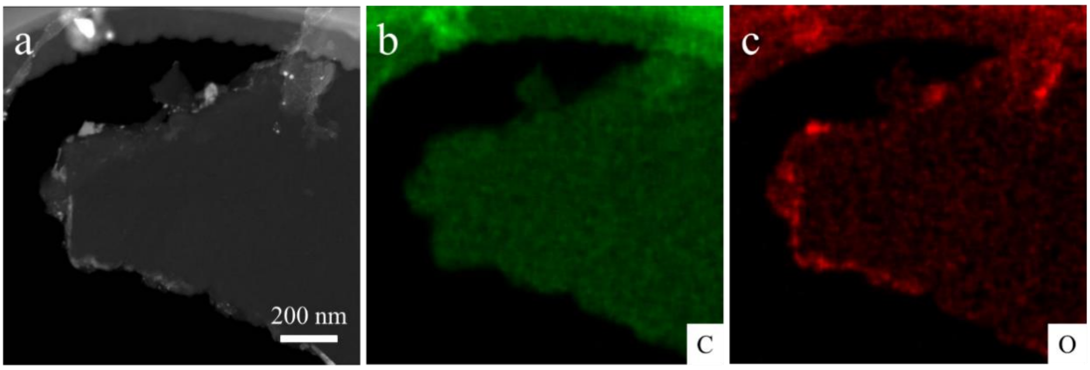

Figure S3. TEM HADDF image and elemental mapping images of the pristine VG. (**a**) HAADF image. (**b**,**c**) Mapping images of different elements C and O existing in the sample.

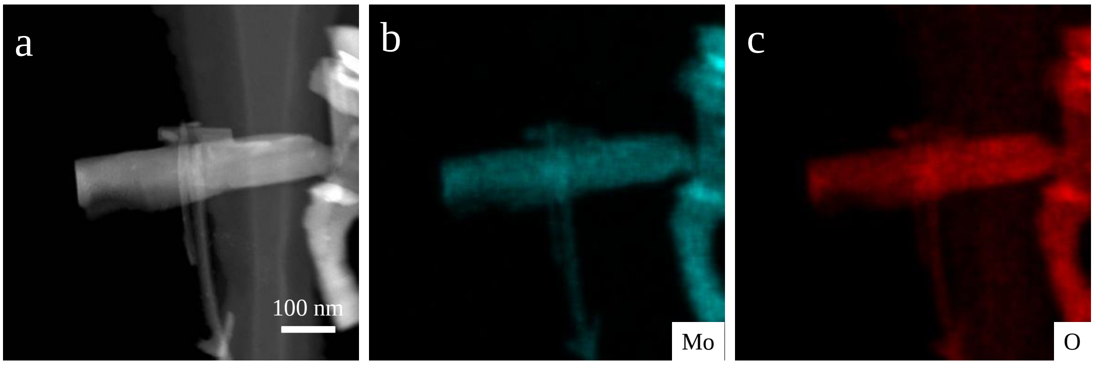

Figure S4. TEM HADDF image and elemental mapping images of the pristine MoO_3 nanosheet. (**a**) HAADF image. (**b**,**c**) Mapping images of different elements Mo and O existing in the sample**.**

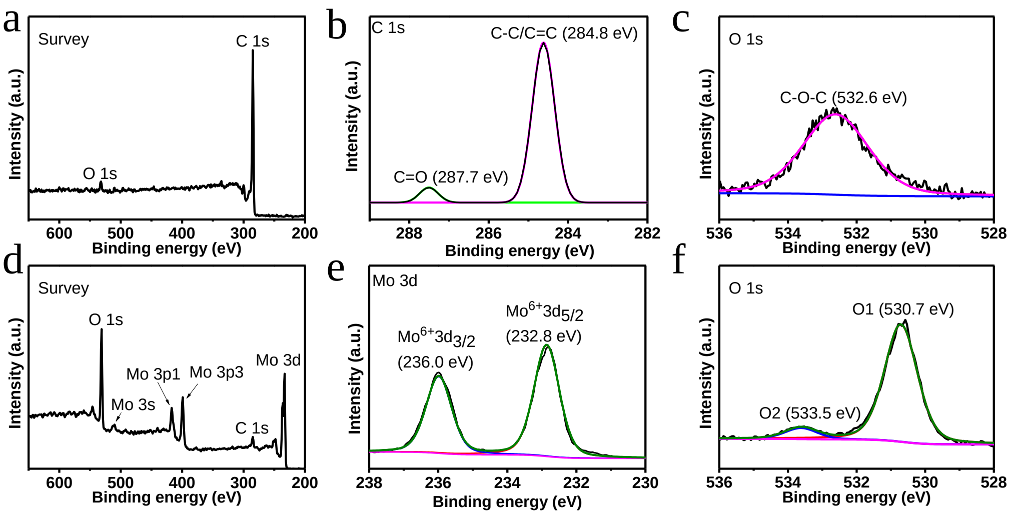

Figure S5. XPS characterizations of the pristine VGs and MoO_3 nanosheets. (**a**) Wide-scanning survey XPS spectrum of the VGs. (**b**,**c**) High-resolution XPS spectra of C 1s and O 1s. (**d**) Wide-scanning survey XPS spectrum of the MoO_3 nanosheets. (**e**,**f**) High-resolution XPS spectra of Mo 3d and O 1s.
